# The Fate of Speckled Protein 100 (Sp100) During Herpesviruses Infection

**DOI:** 10.3389/fcimb.2020.607526

**Published:** 2021-02-01

**Authors:** Mila Collados Rodríguez

**Affiliations:** MRC-University of Glasgow Centre for Virus Research, Glasgow, United Kingdom

**Keywords:** Sp100, herpesviruses, PML-NB, ISG, epigenetics, immunity

## Abstract

The constitutive expression of Speckled-100 (Sp100) is known to restrict the replication of many clinically important DNA viruses. This pre-existing (intrinsic) immune defense to virus infection can be further upregulated upon interferon (IFN) stimulation as a component of the innate immune response. In humans, Sp100 is encoded by a single gene locus, which can produce alternatively spliced isoforms. The widely studied Sp100A, Sp100B, Sp100C and Sp100HMG have functions associated with the transcriptional regulation of viral and cellular chromatin, either directly through their characteristic DNA-binding domains, or indirectly through post-translational modification (PTM) and associated protein interaction networks. Sp100 isoforms are resident component proteins of promyelocytic leukemia-nuclear bodies (PML-NBs), dynamic nuclear sub-structures which regulate host immune defenses against many pathogens. In the case of human herpesviruses, multiple protein antagonists are expressed to relieve viral DNA genome transcriptional silencing imposed by PML-NB and Sp100-derived proteinaceous structures, thereby stimulating viral propagation, pathogenesis, and transmission to new hosts. This review details how different Sp100 isoforms are manipulated during herpesviruses HSV1, VZV, HCMV, EBV, and KSHV infection, identifying gaps in our current knowledge, and highlighting future areas of research.

## Introduction

Speckled 100 kDa protein (Sp100) was identified using autoantibodies from patients suffering from primary biliary cirrhosis autoimmune disease (Szostecki et al., 1987; Szostecki et al., 1990). The ‘speckled’ nuclear distribution of Sp100 predominantly colocalizes with promyelocytic leukemia-nuclear bodies (PML-NBs) (Sternsdorf et al., 1995). Scaffolded by PML (TRIM19), these dynamic nuclear substructures regulate important cellular processes: genome stability, alternative lengthening of telomeres, epigenetic regulation of chromatin, antiproliferation, senescence, apoptosis and antiviral immunity (Gurrieri et al., 2004; Bernardi and Pandolfi, 2007; Scherer and Stamminger, 2016). This range of functions is accomplished by alternatively spliced PML isoforms (Condemine et al., 2006), and its extensive network of protein interactions, some of which are mediated by PML SUMO modification (Van Damme et al., 2010). The post-translation modification (PTM) of proteins by SUMO is common in proteins that harbor a SUMO consensus motif (SCM) [reviewed in (Celen and Sahin, 2020)]. PML and Sp100 have been found to be mono- and poly-SUMOylated (Sternsdorf et al., 1997; Lang et al., 2010; Maarifi et al., 2015). This SUMO “code” is recognized by SUMO interacting motifs (SIMs) present in a variety of cellular proteins known to associate with PML-NBs (Hecker et al., 2006), with SUMO-SIM interactions playing a key role in PML-NB formation and stability (Zhong et al., 2000; Shen et al., 2006; Bernardi and Pandolfi, 2007).

In the following subsections, the domain composition of Sp100 isoforms is detailed, highlighting a role as epigenetic factor that may be independent of PML and PML-NB, and it is especially evident upon herpesviruses infection (see below).

### Protein Architecture of Sp100 Isoforms

**Figure 1:**
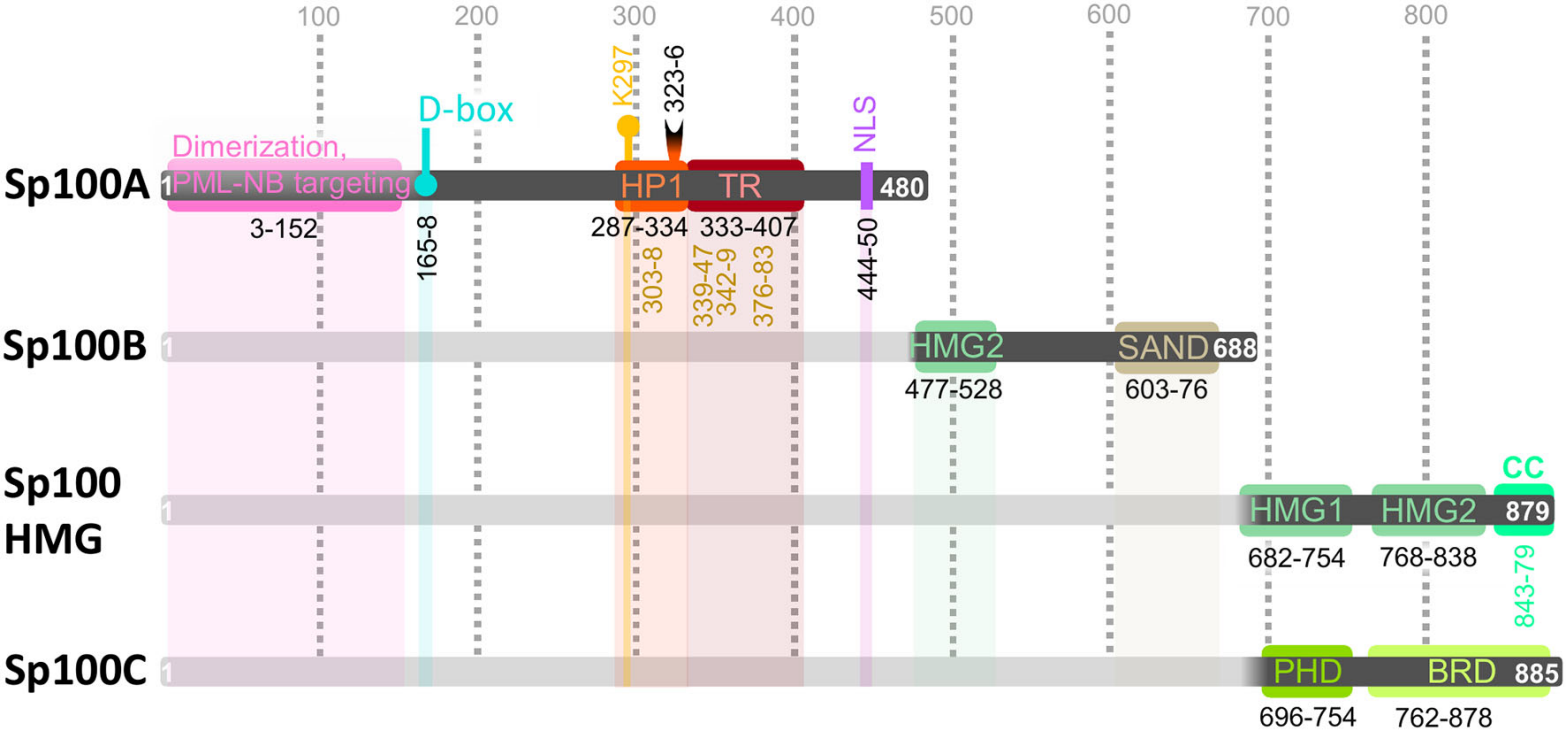
Sp100 isoform domain composition. Sp100A/B/C/high mobility group (HMG) share domain architecture within their first 477 amino acid (aa) residues: dimerization and promyelocytic leukemia-nuclear body (PML-NB) targeting (aa 3–152, pink), destruction-box (D-box, aa 165–168, teal); HP1 interacting region (aa 287–334, orange) encompassing a SUMO consensus motif (SCM) with Lys297 SUMO modification (K297, yellow pin) and SUMO-interacting motif (SIM, aa 323–326, black half-moon); trans-activating region (TR, aa 333–407, cherry); autoepitopes are indicated with vertical numbers in ochre below HP1 and TR segments (see EBV section); nuclear localization sequence (NLS, aa 440–450, purple). Sp100B/C/HMG are identical up to aa 685, which includes the high mobility group (HMG) 2 (aa 477–528, fern) and Sp100, AIRE-1, NucP41/75, DEAF-1 (SAND, aa 603-676, sand) DNA-binding domains. Sp100HMG contains two additional HMG (HMG1, aa 682–754; HMG2, aa 768–838) domains and a coiled coil (CC, aa 843–879, mint) domain. Sp100C contains a plant homeodomain (PHD, aa 696-754, green) and bromodomain (BRD, aa 762–878, light green) domain. C-terminal domain features are described in **Table 1**. Numbers indicate the positions of aa with each isoform. UniProt IDs: Sp100A, P23497-2; Sp100B, P23497-3; Sp100C, P23497-4; Sp100HMG, P23497-1. Further details on the Sp100 gene locus (ENSG00000067066) can be found at ENSEMBL^1^.

The Sp100 gene spans nearly 130,000 bp and contains 32 exons that can be alternately spliced into 19 variants. Of the 11 protein-coding isoforms^1^, only four (Sp100A, B, C, and HMG) have been routinely investigated by the scientific community. These isoforms share the Sp100A domain architecture up to the nuclear localization sequence (NLS) (**Figure 1**), in accordance with their predominant nuclear localization. This N-terminus comprises sequences responsible for Sp100 dimerization and PML-NB localization (Sternsdorf et al., 1999; Negorev et al., 2001), a destruction-box (D-box) required for Sp100 proteasomal degradation (Wang et al., 2011), a SCM (Sternsdorf et al., 1999) and a SIM (Knipscheer et al., 2008; Cuchet et al., 2011) in a histone protein 1 (HP1) interaction site (Seeler et al., 1998), and a trans-activating region (TR) (Szostecki et al., 1990; Szostecki et al., 1992; Xie et al., 1993). Whether these features are shared with the remaining seven coding Sp100 isoforms await to be experimentally determined.

**Figure 2:**
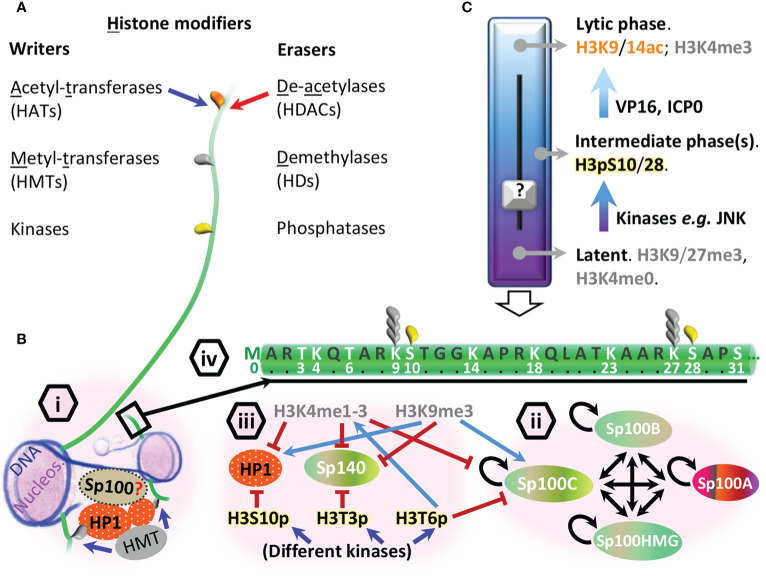
General epigenetic mechanisms influencing chromatin binding properties of Sp100. **(A)** Histone modifier enzymes sorted as “writers” (blue arrow) or “erasers” (red arrow) that influence the acetylation (orange teardrop), methylation (gray teardrop) or phosphorylation (yellow teardrop) post-translational modification (PTM) status of histones exemplified here through H3 tail (green line, not to scale). **(B)** Models for direct (SAND, HMG) and indirect (HP1, BRD, PHD) DNA binding properties of Sp100 isoforms; (i) dimerized HP1s (orange ovals) bind HMT (gray oval) leading to histone H3K9 trimethylation (gray teardrop) of consecutive nucleosomes (lilac). A red question mark indicates whether Sp100B/C/high mobility group (HMG) binds DNA (dark blue string) and/or HP1 as a monomer or as a dimer; (ii) Sp100 can potentially homo- (circular back arrows) and heterodimerize (double head black arrows) depending on the isoform expression profile and subnuclear localization. Sp100 color code refers to **Figure 1** characteristic features; (iii) examples of histone readers’ regulation. H3K4me1-3 inhibits (red flat tip arrows) the binding of Sp100C, Sp140 and HP1 to histone 3, while H3K9me3 promotes (light blue arrows) Sp100C and HP1 binding. Different kinases drive the “phospho-switches” that influence Sp100C, Sp140 and HP1 binding to histone 3 (H3pT3/S10/T6); (iv) histone 3 tail (aa 1–31) highlighting aa and PTMs discussed in the main text associated with the epigenetic silencing of viral DNA and reactivation from latency. **(C)** Viral and epigenetic factors that influence HSV1 transcription; vertical empty arrow pointing down the H3 tail shows an example of post-translational modifications (PTMs) associated with reactivation from latency where the c-Jun N-terminal kinase (JNK) phosphorylates the aa residues next to H3K9/27me3, known as “methyl-phospho switch”, a first step in chromatin relaxation. The participation of Sp100 variants as histone readers in each phase remains to be clearly further detailed (black question mark).

Proteomic studies have shown Sp100 to undergo extensive PTM, including acetylation, phosphorylation, ubiquitination, and SUMOylation (‘ProteomicsDB, Sp100’, 2014). PML-NBs play an important role in the PTM of Sp100, as depletion of PML significantly abrogates the SUMOylation of Sp100 (Everett et al., 2006; Everett et al., 2008; Tavalai et al., 2011) and potentially, other PTMs as phosphorylation (Sternsdorf et al., 1999). The decreased abundance of these PTMs in the absence of PML is probably due to a defect in Sp100 SUMOylation, as depletion of the human E2 SUMO conjugating enzyme (UBC9/UBE2I) leads to similar Sp100 migration patterns in immunoblots (Boutell et al., 2011). It is likely, therefore, that PML mediates, either directly or indirectly, the PTM of Sp100; indeed, PML has been shown to have SUMO E3 ligase activity and to regulate the SUMOylation of a number of PML-NB proteins, including p53, MDM2, Daxx, and c-jun (Quimby et al., 2006). SUMOylation of Sp100 stabilizes its interaction with the C-terminal chromoshadow domain (CSD) present in HP1 proteins (HP1α/CBX5, HP1β/CBX1 and HP1γ/CBX3) (Seeler et al., 1998), but their intermolecular details remain to be fully defined. HP1 can dimerize through CSD domains, creating a platform for histone methyltransferases (HMTs, “histone writer enzymes”, **Figure 2A**) to tri-methylate Lys 9 of histone H3 tails (H3K9me3) where the N-terminal chromo domain (CD) of HP1 binds (Yamamoto and Sonoda, 2003; Larson and Narlikar, 2018), enabling HP1 dimers to bridge consecutive H3 di-nucleosomes (Machida et al., 2018; Kumar and Kono, 2020) (**Figure 2Bi**). HP1 and histones (H2A, H2B, H3, H4, and their respective variants), are examples of the chromatin protein fraction; chromatin is composed of DNA and directly or indirectly associated proteins which compact it in different degrees. Cellular DNA is coiled around nucleosomes formed by histones whose protruding N-terminal tails are subjected to dynamic PTMs (including methylation, acetylation, phosphorylation, ubiquitination and SUMOylation, among others) by histone “writer” and “eraser” enzymes (**Figure 2A**) (Bannister and Kouzarides, 2011); combinations of histone PTMs create chromatin activating or repressive “histone codes” that are interpreted by histone “readers” to regulate transcription (Strahl and Allis, 2000). Such histone modifications are the basis of epigenetics, which lead to cell-type specific and inheritable changes in gene expression without affecting the DNA sequence. The fact that all Sp100 isoforms (Sp100A/B/C/HMG) contain a sequence for PML-NB localization, suggests that some of their functions may be executed at PML-NB; for example, Sp100 crosstalk with chromatin through HP1 interaction may depend on its SUMOylation by PML. Moreover, the impact of homo and heterodimerization of Sp100 (**Figure 2Bii**) on chromatin dynamics is yet to be defined.

While the common N-terminus of Sp100 is mainly involved in protein-protein interactions, features present in its C-terminus directly interact with DNA and histones (**Figure 1**, **Table 1**). Sp100B, C, and HMG contain high mobility group (HMG) and Sp100, AIRE-1, NucP41/75, DEAF-1 (SAND) DNA-binding domains (Guldner et al., 1999; Bottomley et al., 2001). Mechanistically, the Sp100 SAND domain preferentially binds to unmethylated CpG dinucleotides commonly found in foreign DNA (Wilcox et al., 2005; Isaac et al., 2006). HMG binding to DNA opens the minor groove while narrowing the major one, thus bending the DNA to promote nucleosome loading and chromatin remodeling (Thomas, 2001; Malarkey and Churchill, 2012; Lohani and Rajeswari, 2016). Sp100HMG isoform has two additional C-terminal HMG domains (Seeler et al., 1998; Guldner et al., 1999), but their direct participation chromatin modification and assembly warrants additional study. Sp100HMG is also predicted to contain a C-terminal coiled-coil (CC) domain (Kumar et al., 2020), the presence and function of which has yet to be investigated. Sp100C, and Sp100 variant paralogues Sp110, Sp140, and Sp140L which cluster with Sp100 on human chromosome 2, are histone code “readers” since all recognize specific histone tail PTMs through their plant homeodomain (PHD) and bromodomain (BRD) tandem (Dent et al., 1996; Mellor, 2006; Filippakopoulos and Knapp, 2012; Saare et al., 2015; Leu et al., 2018; Zucchelli et al., 2019; Fraschilla and Jeffrey, 2020; Jain et al., 2020). Isothermal titration calorimetry characterization of the Sp100C PHD-BRD tandem peptide (Sp100C_PB_) revealed high affinity for the H3 tails containing the repressive PTMs H3K9me3 and unmethylated H3K4 (H3K4me0), while the chromatin activating marks H3T6p and H3K4me1-3 exclude Sp100C_PB_ binding to H3 tail (**Figure 2Biii**) (Zhang et al., 2016). Overall, this information reveals that Sp100C binding to chromatin can be affected by H3 PTMs. Moreover, *in cellula*, the subnuclear localization of ectopically expressed Sp100C differs from Sp100A and Sp100HMG (Seeler et al., 2001), indicating that the C-terminal domain architecture of each Sp100 isoform also dictates their participation in different chromatin-related processes. Additionally, IFN treatment of epithelial cells has shown to favor the levels of Sp100C mRNA over the other isoforms (Negorev et al., 2009). IFNs are secreted cytokines which activate the assembly of combinatorial STAT complexes that bind to IFN-stimulated response element (ISRE) or to gamma activation site (GAS) at promoters of genes implicated in antiviral defense, known as interferon stimulated genes (ISGs) (Regad and Chelbi-Alix, 2001). Sp100 is an ISG since its promoter contains an ISRE and two GASs binding sites which grant its inducibility by type-I (IFNα, β and κ) and type-II (IFNγ) IFNs in an individual and synergistic way (Grotzinger et al., 1996a; Grotzinger et al., 1996b). Thus, exogenous stimuli can alter the prevailing Sp100 isoform in a given cell type; the molecular details of this shift are still unknown, but splicing driven by Sp100 circular RNA may be involved (Deng et al., 2020).

**Table 1:** Alternatively spliced Sp100 C-terminal domains involved in chromatin regulation.

Domain	Isoform(s)	Function	Reference(s)
HMG2	Sp100B/C/HMG	DNA binding (see below).	(Lehming et al., 1998)
SAND	Sp100B/C/HMG	DNA binding to unmethylated cytosines of CpGs dinucleotides.	(Guldner et al., 1999; Bottomley et al., 2001; Wilcox et al., 2005; Isaac et al., 2006)
HMG1/2	Sp100HMG	Shapes an L composed of three α-helices that bind and open the minor groove of DNA while narrowing the major one thus, bending the DNA and allowing the assembly of nucleosome and other proteins.	(Seeler et al., 1998; Guldner et al., 1999; Thomas, 2001; Malarkey and Churchill, 2012; Newhart et al., 2013; Lohani and Rajeswari, 2016)
CC	Sp100HMG	Unknown.	ELM prediction (Kumar et al., 2020)
PHD	Sp100C	Binds with most affinity to histone 3 unmethylated in Lys4 (H3K4me0) but tri-methylated in Lys9 (H3K9me3).	(Zhang et al., 2016)
BRD	Sp100C	Unclear binding to acetylated Lys (KAc).	(Filippakopoulos and Knapp, 2012; Zhang et al., 2016)

### Further Details of Sp100C in Crosstalk With Chromatin

Other activating H3 PTMs such as mono- (H3K14/18/27ac) or multi acetylation (H3K14/18/23ac), phosphorylations at H3T3p, H3KS10p, or H3K9me3S10p, do not affect Sp100C_PB_ binding to H3 tail; since the Sp100C BRD could not bind H3Kac *in vitro* either, it was indicated that the BRD molecular function is unknown but it was critical to stabilize the Sp100C PHD fold, given the extensive contacts seen in their crystal structure (Zhang et al., 2016). Of note, the PHDs of other proteins, as Sp140, have shown to facilitate BRD SUMOylation and its association with SETDB1, a HMT of H3K9 that promotes gene silencing (Ivanov et al., 2007; Peng and Wysocka, 2008; Garcia-Dominguez et al., 2008; Zucchelli et al., 2019); interestingly, this PTM also weakens the Sp140 PHD binding to H3 tail (**Figure 2Biii**) (Zhang et al., 2016). Future *in vivo* studies may indicate if Sp100C BRD has affinity for any H3Kac residues (**Figure 2Biv**), whether BRD SUMOylation takes place and affects Sp100C binding to H3, and if Sp100 orthologues cooperate as histone code readers.

Sp100C PHD is singular in tolerating H3T3p, since this PTM acts as a “binary switch” on Sp140 by excluding the binding of Sp140 PHD to H3 (Zhang et al., 2016) (**Figure 2Biii**). H3T3 is phosphorylated upon DNA damage (Salzano et al., 2015) and during prophase (Dai et al., 2005), while it is dephosphorylated during anaphase (Dai et al., 2005). Taken together, this suggests that Sp100C would still be bound to H3T3p during these processes, while Sp140 is not. Moreover, it has been reviewed that H3K4me0 and H3K9me3 represent marks to coordinate and maintain DNA methylation memory through mitosis (Hashimoto et al., 2010); it is tempting to speculate about the participation of Sp100C in ensuring this process.

In post mitotic neurons, H3S10 phosphorylation detaches HP1 from H3K9me3 (**Figure 2Biii**) while retaining ATRX (Noh et al., 2015), an ATP-dependent chromatin remodeller which, as Sp100C, requires both H3K9me3 and H3K4me0 to bind H3; ATRX cooperates with HP1 and the H3.3 chaperone Daxx to keep telomeric and pericentromeric chromatin repressed (Eustermann et al., 2011; Clynes et al., 2013). H3.3 is an histone variant loaded onto chromatin independently of DNA synthesis (Tagami et al., 2004) and then maintained by the PML-NB proteins and H3.3 chaperones Daxx/ATRX (Cabral et al., 2018). Since H3K9me3 and H3K4me0 can be read by multiple molecules, the spatio-temporal dynamics of Sp100C binding to H3 respective to other molecules as Sp140, HP1 and ATRX, remains to be determined. Overall, Sp100C PHD binds to repressive chromatin but tolerates activating epigenetic marks, except H3K4me and H3T6p.

Collectively, Sp100A/B/C/HMG isoforms share the N-terminus which allows their dimerization and location at PML-NB, where they can be modified with SUMO to interact with HP1, a chromatin protein. The Sp100B/C/HMG SAND domain links them to typically exogenous unmethylated DNA CpGs, but functions assigned to additional domains have to be directly established for Sp100 *in vivo*. Detailed Sp100C studies have evidenced its predominant transcription upon IFN treatment and its *in vitro* participation in chromatin compaction when H3 tails lack H3T6p and H3K4me. Further Sp100 characterization is key to understand its participation in cellular and viral chromatin regulation.

## Clinical Importance of Herpesviruses and Molecular Basis

Herpesviruses are a large family of double stranded DNA viruses that cause a variety of clinically important diseases on a global scale (**Table 2**). Their success lies their ability of reactivating from a latent state of infection whereby viral DNA (vDNA) transcription in the nucleus of terminally differentiated (nerve or white blood) cells is not enough to generate virus progeny, thereby avoiding immune clearance and thus, keeping a host chronically infected for a lifetime. Consequently, understanding the host defenses that limit the reactivation, propagation, and transmission of herpesviruses is of global importance.

**Table 2:** Clinical relevance of herpesviruses.

Virus	Clinical manifestations and estimates (references)
Herpes simplex virus 1 (HSV1)/HHV1	Blindness, dermatitis, gum diseases, sores in the mouth, nose and genitals, newborn fatal encephalitis (Imbronito et al., 2008; Looker et al., 2017; Marcocci et al., 2020). Considered causative factor of sporadic Alzheimer disease (Cairns et al., 2020). In 2016, the WHO (World Health Organization, 2020) estimated that about 67% of the world’s population below 49 years old had HSV1 infection.
Varicella zoster virus (VZV)/HHV3	Primary infection causes chicken pox/varicella and infectious shingles upon reactivation; during 2008–2011, VZV mortality rate in the US population was estimated to be 0.05 per million, representing an 87% decrease in comparison to prevaccine years (Johnson and Levin, 2020).
Human cytomegalovirus (HCMV)/HVV5	Worldwide seroprevalence of 66%–90% (Zuhair et al., 2019). It affects transplants’ recipients (Schottstedt et al., 2010; Meesing and Razonable, 2018) and is a major cause of congenital disability in children (Davis et al., 2017; Emery and Lazzarotto, 2017).
Epstein-Barr virus (EBV)/HVV4	Mononucleosis and derived lymphomas caused at least 142,000 deaths worldwide in 2010 (Khan and Hashim, 2014; Martinez and Krams, 2017) and a chronic population deterioration resulting from EBV-related autoimmune diseases (Draborg et al., 2016; Balandraud and Roudier, 2018; Trier et al., 2018).
Kaposi’s sarcoma-associated herpesvirus (KSHV)/HVV8	KSHV associated tumorigenesis is responsible for around 55% mortality in South African infected children (Dow et al., 2014), and nearly 20% of deaths in seropositive blood transfusions (Ablashi et al., 2002; Operskalski, 2012).

Primary infection of herpesviruses starts with their binding to a cell surface, followed by nucleocapsid entering the cytoplasm, transport and attachment to the nuclear pore to release the viral genome into the nucleus; although vDNA is ejected into the nucleoplasm as a naked molecule, viral tegument proteins characteristic of each virus subfamily may access the nucleus with different efficiencies depending on the cell type infected, influencing the subsequent events in diverse ways [reviewed by (Full and Ensser, 2019)]. In parallel, the equilibrium of pro- and antiviral host factors intrinsic (pre-existing) to each cell type, ready to associate with this foreign nucleic acid and viral tegument proteins, dictates whether the infection progresses to lytic replication or remains latent.

When primary infection takes place in epithelial cells (*e.g.* in mucosa), herpesviruses typically initiate a lytic cycle of replication through the coordinated expression of immediate-early (IE), early (E), and late (L) genes, which sequentially contribute to the inactivation of host immune defenses, stimulation of viral gene expression, vDNA replication and virion assembly, respectively (Gruffat et al., 2016). In contrast, infection of terminally differentiated cell types is prone to latency because herpesviruses generally fail to establish this temporal cascade of viral gene expression. This once perceived binary (lytic or latent) behavior has recently been shown to be more heterogeneous, with patterns of viral gene expression dependent on cell type, genome copy number, and degree of pre-immune stimulation prior to infection (Knipe and Cliffe, 2008; Suzich and Cliffe, 2018). One of the intrinsic immunity barriers herpesviruses have to counteract or exploit upon vDNA release into the nucleus is chromatinization and epigenetic modulation (Knipe et al., 2013). Moreover, cellular stress can affect vDNA chromatinization, partially or fully reactivating viral transcription programs with variable production of progeny virions (reviewed by (Weidner-Glunde et al., 2020)). For example, neuronal stress promotes the phosphorylation of H3S10/28 at vDNA promoters by c-Jun N-terminal kinase (JNK) (**Figures 2Biv, C**), allowing viral gene expression even when adjacent H3K9/27me3 repressive marks are present (Cliffe et al., 2015). Despite of Sp100 is unaffected by H3pS10 epigenetic switch (see introduction), expression of further herpesviruses proteins neutralize specific antiviral immunity factors as Sp100, triggering periodic reactivation of viral latent pools, leading to *de novo* virus replication, virus progeny production and transmission to new hosts.

## The Spatiotemporal Journey of Sp100 Against Herpesviruses

**Figure 3:**
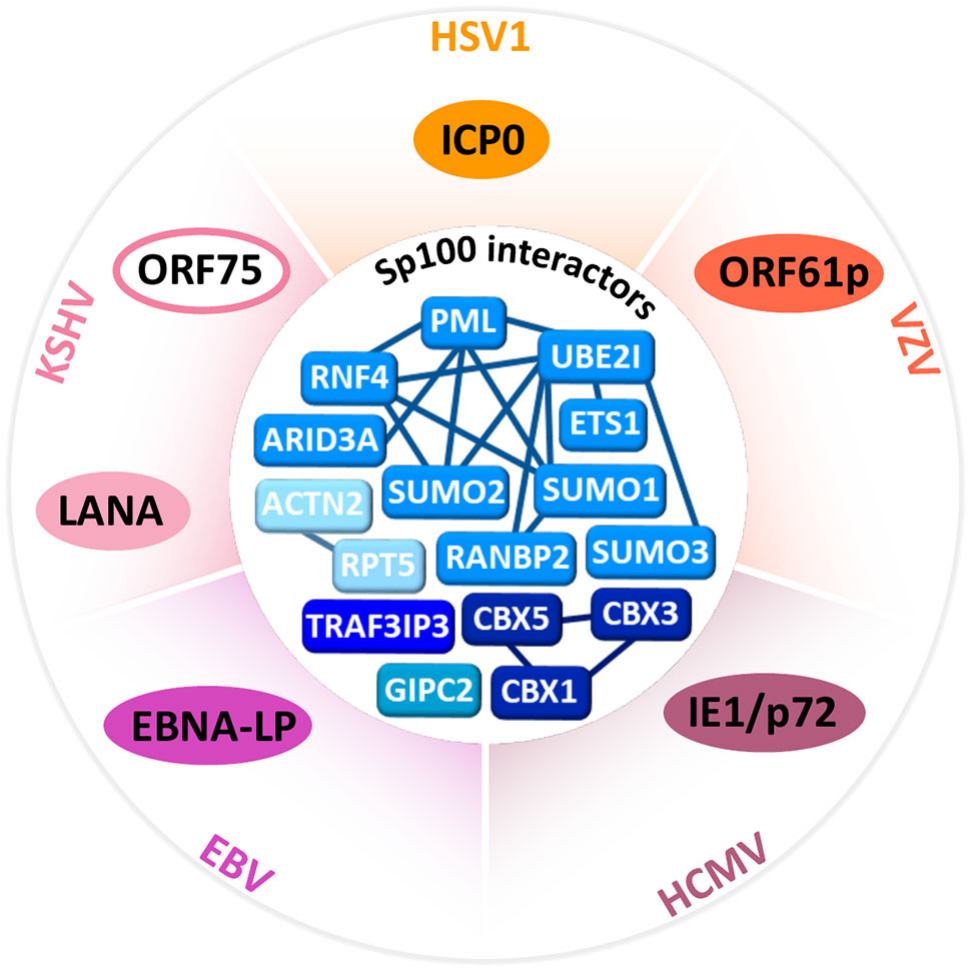
Sp100 interactors and herpesvirus counteractors. Outer circle, herpesviruses proteins (HSV1, ICP0; VZV, ORF61p; HCMV, IE1/p72; EBV, EBNA-LP; KSHV, LANA, and ORF75) that antagonize Sp100. Inner circle, network of Sp100 related protein interactions likely to be disrupted during herpesvirus infection. Clustered networks of known Sp100 interactors shaded in blue. Interactions retrieved from BioGRID (Stark et al., 2006), minimum experimental evidence from two independent studies.

This section focuses on the antiviral properties of the Sp100 variants and how they are hijacked from the outset of infection by herpesviruses factors; **Figure 3** summarizes these viral proteins counteracting Sp100 and indirectly affecting its core network of cellular protein partners. Since different aspects of epigenetic modulation of herpesviruses have been characteristically studied for each family (Knipe et al., 2013), only these known to affect Sp100 fate are highlighted, introduced in the HSV1 section, and subsequently referred.

### Herpes Simplex Virus 1

When HSV1 infection progresses to lytic replication, parental vDNA is not associated to typical nucleosome units but to proteinaceous structures (Muggeridge and Fraser, 1986; Deshmane and Fraser, 1989). This incomplete vDNA chromatinization may result from the blocking of histone loading into naked DNA by VP22, the most abundant tegument protein (van Leeuwen et al., 2003). In fact, H3.3 has been observed to accumulate juxtaposed but unincorporated to vDNA into PML-NB, which could be the aforementioned proteinaceous structures detected at the initial and lytic phases of infection (Conn et al., 2013). Accordingly, PML-NB are known to act as protein depots (Negorev and Maul, 2001), as well as to assemble onto and entrap incoming vDNA (Alandijany et al., 2018). PML-NB entrapped vDNA has been shown to lack H3K4me2 but to be H3.3 decorated with H3K9me3 (Cabral et al., 2018; Cohen et al., 2018), advancing Sp100C presence. Moreover, experiments studying the recruitment of PML-NB proteins to a transgene array as a model for foreign DNA invasion, showed that when UBC9 was depleted, neither Daxx, PML nor H3.3 accumulated onto the array; hence, their accumulation onto invading DNA depends on SUMOylation (Shastrula et al., 2019), an enzymatic activity enriched at PML-NB (see introduction). However, whether SUMOylation is also required for Sp100 accumulation at vDNA is unclear but seems to be isoform-specific, since Sp100 spliced variants have commonalities but also differences (**Figure 1**), which may account for their divergent spatio-temporal behavior: Sp100A has been shown to enhance the expression of the IE infected cell protein-0 (ICP0) promoter, while it is repressed by Sp100 isoforms containing a SAND domain (Sp100B/C/HMG), unless this domain is mutated (Negorev et al., 2006); similarly, Daxx, ATRX and Sp100B/C/HMG repress a CMV promoter reporter while withdrawing Sp100A-driven chromatin decondensation (Seeler et al., 1998; Newhart et al., 2012; Newhart et al., 2013). Taken together, Sp100B/C/HMG cause repression of transcription while Sp100A is activating in a mutually exclusive way.

In turn, this differential transcriptional behavior of Sp100B/C/HMG as opposed to Sp100A is distinctly modulated by ICP0 since when HSV1 ICP0 was included in the CMV promoter reporter, Sp100A presence augmented at this CMV promoter reporter independently of its SUMOylation status, Daxx or ATRX, while Sp100B/C/HMG were degraded (Newhart et al., 2013). It is known that challenging epithelial cells with HSV1 depletes Sp100 SUMOylated isoforms (Everett et al., 2009), as occurs when UBC9 or PML are silenced by shRNA (Everett et al., 2006). Thus, Sp100 degradation could either be a direct target of ICP0 (Perusina Lanfranca et al., 2013), a viral E3 Ub ligase which preferentially targets SUMOylated proteins for proteasomal degradation [(Boutell et al., 2011), reviewed by (Boutell and Everett, 2013; Rodriguez et al., 2020)], or occur as an indirect consequence of PML disposal by ICP0 (Tavalai and Stamminger, 2009). In any case, dismantling of PML-NB by ICP0 or by PML shRNA silencing has no effect on Sp100A (Everett et al., 2006; Everett et al., 2009), advancing that Sp100A transactivating properties may be exploited by HSV1 (see below).

Interestingly, Sp100A SIM deletion has shown that in ΔICP0 HSV1 infected cells, Sp100A can prescind from its SUMOylation but requires its SIM to appear as nuclear puncta even in the absence of PML (Cuchet et al., 2011; Cuchet-Lourenco et al., 2011); this suggests that Sp100, as PML, may scaffold factors on its own. In order to study the participation of Sp100 in restricting HSV1 infection, mutant viruses lacking ICP0 (ΔICP0) or carrying mutations in its catalytic RING-finger domain responsible of its E3 Ub ligase activity are routinely used to keep PML-NBs intact. Independent or combined depletion of PML and Sp100 shows that their effects in restricting ΔICP0 HSV1 are additive but partial (Everett et al., 2006; Everett et al., 2008; Glass and Everett, 2013). This indicates that both factors have antiviral properties on their own, which are enhanced by their cooperation, but they do not fully restore the ΔICP0 effects to WT HSV1 levels because additional proteins participate in counteracting ICP0. Indeed, the chromatin regulators Daxx, ATRX and MORC3 can be still recruited to incoming viral genomes when both Sp100 and PML are silenced (Everett et al., 2008; Lukashchuk and Everett, 2010; Cuchet et al., 2011; Sloan et al., 2016). MORC3 dimerizes to acts as ATPase when its histone recognition CW domain binds H3K4me3 (Zhang et al., 2019), forming MORC3-NB; speculatively, the ATPase function of MORC3 may fuel chromatin remodeling enzymes (Vignali et al., 2000). Complementarily, silencing MORC3 by shRNA impedes PML, Sp100 and Daxx appearing as puncta before the emergence of replication compartments (RCs) visualized with ICP4; however, once RCs appear, they are PML free but Sp100 is still associated to them (Everett et al., 2006; Everett et al., 2008; Lukashchuk and Everett, 2010; Cuchet et al., 2011; Sloan et al., 2016). This suggests that the recruitment of PML, Sp100 and Daxx to parental HSV1 DNA is orchestrated by MORC3 to form PML-NBs on the one hand, and on the other hand, that a portion of Sp100 associates to ICP4 replication centers independently of MORC3 when PML-NBs have been dismantled by ICP0. Collectively, this indicates that the cell can recruit energy fueling enzymes to ensure subsequent viral chromatin remodeling but, contrary to Sp100A, they are targeted by ICP0.

HSV1 ΔICP0 parental genome have limited gene expression as it remains entrapped by PML-NBs upon infection of the cell nucleus (Everett and Murray, 2005; Alandijany et al., 2018). This is so unless the copy numbers of HSV1 ΔICP0 saturate the intrinsic defenses that PML-NB represent, escaping vDNA entrapment and leading to its replication (Alandijany et al., 2018; McFarlane et al., 2019). Viral replication triggers cytokine signaling which leads to the PML and Sp100-dependent accumulation of the H3.3 chaperone HIRA at pre-existing PML-NB, since shRNA silencing of either PML or Sp100 abrogates HIRA accumulation at PML-NBs (Alandijany et al., 2018; McFarlane et al., 2019). HIRA binds ISGs loci to promote their transcriptional upregulation, further stimulating the innate immune defenses upon HSV1 infection (Alandijany et al., 2018; McFarlane et al., 2019); in this way, undisrupted PML-NB are able to induce an IFN response upon HSV1 ΔICP0 infection.

Another HSV1 factor accessing epithelial cells nucleoplasm is VP16, which transactivates IE genes upon recruiting coactivators to their promoters; one of these coactivators, host cell factor-1 (HCF-1), has been reviewed to associate with H3K4 HMTs to ensure H3K4me3 presence (Kristie et al., 2010; Vogel and Kristie, 2013). This chromatin activation mark would exclude Sp100C, Sp140 and HP1 from H3 (see introduction, **Figure 2Biii**) however, this remains to be assessed. In contrast, HSV1 cannot take advantage of VP16 and VP22 in neurons since these tegument proteins dissociate from the capsid before it reaches the nucleus (Aggarwal et al., 2012), a process favoring latency establishment in these cells over epithelial ones. Nevertheless, literature studying the participation of Sp100 during HSV1 latency establishment and maintenance is scarce (Everett et al., 2007; Cohen et al., 2018), and key questions as whether neurons depleted for Sp100 establish latency are still unanswered.

In conclusion, the chromatin repressive Sp100 SUMOylated isoforms seem to be required to entrap parental vDNA in cooperation with other SUMOylated chromatin-associated factors, but they are (directly or indirectly) targeted by HSV1 ICP0 and possibly, indirectly counteracted by VP16 and VP22. In contrast, the remaining unSUMOylated Sp100A has chromatin activating properties and can form NB independently of PML, which may represent a favorable environment for viral replication. Since Sp100A domain architecture is present in most of the other isoforms, it would be challenging to specifically target it with drugs however, when PML-NB are dismantled by ICP0, Sp100A seems to harbor differential PTM which may make it drug-amenable.

### Varicella Zoster Virus

The above described Sp100 isoforms’ dynamics on chromatin also apply to herpesviruses other than HSV1 however, they are affected at a different extent by ICP0 homologs. The ICP0 VZV homolog VICP0/ORF61p only targets Sp100 out of the PML-NB components (Walters et al., 2010). ORF61p also harbors a RING-finger and SIMs to function as E3 Ub ligase on SUMOylated targets however, ORF61p lacks sequences required for binding to the host deubiquitinase USP7, which protects ICP0 from auto-ubiquitination and proteasome-mediated degradation (Kyratsous and Silverstein, 2009). As a result, ORF61p turnover by proteasomal degradation is quicker than for ICP0, making ORF61p more unstable than its HSV1 homolog (Everett et al., 2010). Therefore, IE kinetic studies comparing ORF61p to ICP0 in an HSV1 background evidence that ORF61p incompletely substitutes for ICP0; contrary to ICP0, ORF61p only reduces Sp100 levels without targeting PML (Kyratsous et al., 2009). Consequently, VZV infected cells still harbor PML-NB capable enough of sustaining an IFN response, which further increases Sp100 and PML levels and allows PML to SUMOylate Sp100; in agreement, late kinetic studies by immunoblot show a predominant increase of SUMOylated Sp100A (Kyratsous and Silverstein, 2009). At this late time point, the corresponding immunofluorescence images of cells display a granulated distribution of Sp100 in the nucleoplasm, concomitant with a high abundance of ORF61p (Kyratsous and Silverstein, 2009), but point mutations on its RING domain change the dispersed pattern to nuclear puncta, still colocalizing with Sp100 (Walters et al., 2010); the significance of this observation in the context of VZV life cycle requires further experimental assessment. In summary, Sp100 levels are reduced by VZV ORF61p from the onset of infection but since VZV does not completely disrupt PML-NBs, IFN-response can rise Sp100 protein levels, which are kept dispersed in the nucleoplasm by ORF61p. Whether Sp100 degradation or dispersion also facilitates initiation of VZV lytic replication *per se*, remains to be investigated.

### Human Cytomegalovirus

The role of immunity factors restricting HCMV infection, with Sp100 as PML-NB component, has been reviewed elsewhere (Rossini et al., 2012; Landolfo et al., 2016). As opposed to Sp100 merely being used as a PML-NB marker assumed to behave as PML (Ahn and Hayward, 1997; Sourvinos et al., 2007), this section focuses on Sp100 isoforms during HCMV infection. After parental HCMV genomes enters the nucleus, alternative splicing of a sole HCMV IE transcript results in two proteins: IE1/p72 and IE2/p86 (Stenberg et al., 1985). IE1 initially co-localizes at PML-NBs and then gradually disperses them through the nucleoplasm (Korioth et al., 1996). More in detail, IE1 has been shown to interact with the Sp100 N-terminal dimerization domain, as deletion of the corresponding 3–152 aa abrogates their association, and infection with mutant HCMV lacking IE1 does not cause loss of Sp100 (Kim et al., 2011). In turn, Sp100 depletion favors IE1 expression (Kim et al., 2011; Ashley et al., 2017); individual depletion of PML, Sp100 or Daxx showed that each factor was restrictive on its own (Ashley et al., 2017), combined depletion of Sp100/PML or Sp100/Daxx further enhanced HCMV gene expression initiation (Adler et al., 2011), and combined depletion of all PML/Sp100/Daxx were more permissive to HCMV infection (Ashley et al., 2017), resembling the above discussed intrinsic immunity factor’s dynamics against HSV1. Similarly, all PML/Sp100/Daxx depletion also reduces the restriction of HCMV by IFNβ, visualized through IE1 presence and plaque assay upon IFNβ treatment (Ashley et al., 2017), indicating that these PML-NB components mediate the IFN response against HCMV. IE1 co-transfection with each Sp100 isoform was shown to reduce their SUMOylation (Tavalai et al., 2011); this effect is especially evident for Sp100A (Dimitropoulou et al., 2010; Tavalai et al., 2011), but unSUMOylated Sp100A levels also decrease at later times post HCMV infection (Tavalai et al., 2011). Even more, IE1 gets SUMOylated while driving the deSUMOylation of Sp100 and PML (Muller and Dejean, 1999). PML function as E3 SUMO ligase onto IE1 has been evidenced since PML RING domain mutants fail to SUMOylate IE1 (Reuter et al., 2017); this further supports the possibility of PML directly SUMOylating Sp100, indicating that part of the antiviral effect of Sp100 depends on PML. Overall, Sp100 disruption by HCMV IE1 seems to affect all isoforms, although at slower kinetics than HSV1 ICP0.

Furthermore, the promoter of the IE1/2 transcript, the major immediate early promoter (MIEP) has been shown to be repressed by histone deacetylase 3 (HDAC3) and HP1 in peripheral blood monocytes (Murphy et al., 2002), where HCMV establishes latency; this suggests that sustained histone deacetylation may allow histone methyltransferases (HMTs) to lock MIEP chromatin in a repressive state characteristic of a latent state of infection. However, the participation of Sp100 in latency establishment has been excluded using THP-1 derived macrophages partially depleted for either Sp100, Daxx or PML; intriguingly, partial Daxx depletion was enough to increase Sp100A levels similarly to undifferentiated THP-1 monocytes, especially unSUMOylated Sp100A ones, while PML had no effect on Sp100 levels (Wagenknecht et al., 2015), contrasting with previous reports. A more robust cellular KO background may consolidate or rule out the implication of Sp100 isoforms and additional factors in HCMV latency establishment and cell identity.

### Epstein–Barr Virus

Twenty years ago it was stated that latent EBV episomes are tethered to cell chromosomes away from PML-NB (Bell et al., 2000), contrasting with the later observation of the IE EBV protein EBNA-LP colocalizing with Sp100 at PML-NB in EBV immortalized lymphoblastoid cell lines (Ling et al., 2005). Using transfection assays, EBNA-LP was shown to disperse Sp100 from PML-NB by interacting with the Sp100 PML-NB targeting domain (Ling et al., 2005); dispersion by either EBNA-LP or by deleting the Sp100 PML-NB targeting domain, dissociates Sp100 away from PML-NB allowing the viral oncoprotein EBNA2 to act as a transcriptional activator, even in the absence of EBNA-LP (Ling et al., 2005). Interestingly, for this process to occur, the Sp100 HP1 interacting domain was required to be intact, but not the SCM embedded on it, since K297R modified Sp100A still activates EBNA2 even upon IFNβ pretreatment (Ling et al., 2005; Echendu and Ling, 2008). Hence, high resolution microscopy studies looking at whether Sp100 isoforms associate at some point of the viral cycle with EBV episomes await to be accomplished. Moreover, whether Sp100 SIM, which is also embedded in its HP1 interacting domain, is also required for the effective EBV subversion of innate immunity, which culminates in lytic reactivation remains to be addressed. Thus, EBV EBNA-LP is equivalent to HSV1 ICP0 in the sense of dispersing proteins from PML-NB and opens new questions as how different herpesvirus co-infections may affect each other’s lytic replication upon reactivation. In fact, EBNA-LP increases HSV1 ΔICP0 replication, depletes Sp100 SUMOylated isoforms and causes a Sp100 mobility shift (Lu et al., 2016) characteristic of lack of localization at PML-NB. To sum up, EBNA-LP selectively binds to the PML-NB targeting domain of Sp100 abrogating its PML-NB localization, but details of the fate of specific Sp100 isoforms are unknown.

The significance of the above described Sp100 overexpression studies establishes parallelisms to clinical pathological cases of Sp100 overexpression and nucleoplasm delocalization which may favor the chance of generating Sp100 autoantibodies by molecular mimicry. EBV has been linked to PML-NB associated autoimmune diseases since two viral proteins share autoepitopes with Sp100 [aa 296–311 and aa 332–351 in ochre, **Figure 1**; (Xie and Snyder, 1995)]. These epitopes partly coincide with the ones described for 20-30% of patient’s sera with biliary cirrhosis [aa 303–308 and aa 339–347 in ochre, **Figure 1**; (Bluthner et al., 1999)]. Such Sp100 antigenic regions flank its SIM and fall along the HP1 interacting region and in its TR. Consequently, it can be envisaged that autoantibodies against SIM, HP1 or TR Sp100 regions may sterically impede the interaction of Sp100 SIM with SUMO conjugated to other proteins, the interaction between HP1 proteins and Sp100, and the Sp100 trans-activating capabilities, respectively. Knowing Sp100-derived autoepitopes creates a chance for pharmacological intervention tailored to different Sp100 regions to counteract the EBV targeting of Sp100 at molecular and humoral levels.

### Kaposi’s Sarcoma-Associated Herpesvirus

KSHV research comprising Sp100 is mostly related to the establishment of latency, which depends on translating the latency associated nuclear antigen (LANA) (Campbell and Izumiya, 2012), as well as reactivation, which occurs when the tegument protein ORF75 disperses Sp100 from PML-NB (Full et al., 2014). During primary infection, ORF75 has no effect on Sp100 and PML; these PML-NB components restrict KSHV, as their individual silencing allow viral proteins expression (Full et al., 2014). As a result of KSHV infection there is an IFN-mediated increase of Sp100 levels however, the viral encoded E3 SUMO ligase LANA converts the Sp100 soluble pool residing in the nucleoplasm and cellular chromatin into an insoluble one by inducing Sp100 SUMOylation and storage into the insoluble nuclear matrix, presumably corresponding to PML-NB or to another fraction (Gunther et al., 2014). Sp100 insolubilization allows the parental vDNA to establish latency and eventually reactivate by acquiring the H3K27me3 repressive mark (Gunther and Grundhoff, 2010), characteristic of facultative chromatin, which was shown to be favored by silencing Sp100 (Gunther et al., 2014). The Sp100 insolubilization by LANA seems to be unique of KSHV as other γ-herpesviruses as EBV maintain Sp100 soluble during latency (Gunther et al., 2014); nevertheless, similar analysis of the insoluble fractions for other herpesviruses are sparse across the literature. Collectively, undisrupted PML-NB allow IFN induction upon KSHV infection, increasing Sp100 levels but maintaining them in an insoluble form by LANA-mediated SUMOylation; this is concomitant with the acquisition of the repressive H3K27me3 mark, characteristic of latency, by parental vDNA.

## Conclusions and Future Perspectives

Herpesviruses attain a latent state of infection with periodic complete or incomplete reactivation which cause an underestimated quality of life deterioration. Since there is no cure, understanding how the cocktail of Sp100 isoforms are counteracted in each cell type as intrinsic and innate immunity factors by herpesviral proteins may help in its development. Recapitulating, the Sp100B/C/HMG isoforms, and likely other Sp-family members, may sense invading vDNA and coordinate the assembly of H3 and chromatin repressive marks at PML-NB SUMOylation and epigenetic hubs. However, herpesviruses can directly counteract the Sp100 role in viral epigenetics, as well as indirectly by dismantling PML-NB; HSV1 and HCMV IE proteins disrupt PML-NB, while VZV, EBV and KSHV IE proteins selectively target Sp100 thus, PML-NB can still induce an IFN response which leads to vDNA repression, promoting latency establishment. Much remains to be discovered concerning the molecular details leading to latency and the intermediate steps leading to reactivation. This review has highlighted the need to better understand cell-specific dynamics of Sp100 isoforms, characterize further ones, as well as their individual features interplaying with other chromatin factors, framing areas for pharmacological exploration.

## Author Contributions

MCR wrote, edited the manuscript, and prepared the figures.

## Funding

This work was supported by the Medical Research Council (https://mrc.ukri.org) grant MC_UU_12014/5 (to Dr Chris Boutell).

## Conflict of Interest

The author declares that the research was conducted in the absence of any commercial or financial relationships that could be construed as a potential conflict of interest.

## Abbreviations

aa, amino acid residues; ATRX, alpha-thalassemia/mental retardation X-linked; bp, base pairs; Daxx, death domain associated protein; EBNA-LP, Epstein-Barr virus nuclear antigen-leader protein; HIRA, histone cell cycle regulator A; HMG, high mobility group; Kbp, kilobasepairs; MORC3, microrchidia 3; SETDB1, SET domain bifurcated histone lysine methyltransferase 1; STAT, signal transducer and activator of transcription; SUMO, small ubiquitin (Ub) modifier; UBC9, Ub conjugating enzyme 9; UBE2I, Ub conjugating enzyme E2I; VP, viral protein; WHO, World Health Organization.
